# Acupoint Massage Therapy Alters the Composition of Gut Microbiome in Functional Constipation Patients

**DOI:** 10.1155/2021/1416236

**Published:** 2021-01-12

**Authors:** Hui Chen, Pei-Shan Tan, Chun-Ping Li, Bi-Zhen Chen, Yu-Qin Xu, Yan-Qin He, Xiao Ke

**Affiliations:** ^1^Department of the Key Specialty of Spleen and Stomach, The Second People's Hospital Affiliated to Fujian University of Traditional Chinese Medicine, Fuzhou, China; ^2^Department of the Internal Medicine, Fuzhou Economic and Technological Development Zone Hospital, Fuzhou, China; ^3^Department of the Infection-Control, The Second People's Hospital Affiliated to Fujian University of Traditional Chinese Medicine Hospital, Fuzhou, China; ^4^Department of the Personnel Office, The Second People's Hospital Affiliated to Fujian University of Traditional Chinese Medicine Hospital, Fuzhou, China

## Abstract

**Results:**

Results showed the overall structure of gut microbiome has no significant difference between experimental and control groups. In the genus level, the abundance of *Pseudobutyrivibrio* and *Ruminiclostridium* is higher in the experiment group than in the control, whereas that of *Fusicatenibacter* is less. The 16S KEGG function prediction suggested that Parkinson disease, retinol metabolism, and arachidonic acid metabolism could explain the biological function of different gut microbiome. Furthermore, cytokines in the serum showed a correlation with the abundance of *Pseudobutyrivibrio* in CFC.

**Conclusion:**

AMT could change the composition of gut microbiome which is associated with cytokines in CFC patients.

## 1. Introduction

Constipation is a high-incident clinical disease of the digestive system globally [1]. Its principal manifestations are fecal frequency reduction, dry stool, and/or difficulty in defecation. Depending on the different causes of constipation, it can be divided into organic constipation and functional constipation [2]. Most of the chronic constipation patients have chronic functional constipation (CFC). The quality of CFC patient life is significantly lower than that of the healthy people. It can be expected to result in many diseases such as cardiovascular and cerebrovascular diseases and colon cancer and do great harm to the human body.

At present, there is no ideal treatment for CFC; clinical therapy mainly relies on drug treatment [3]. The commonly used drugs include volume laxatives (lactulose and polyethylene glycol), irritant laxatives (bisacodine and senna leaf) and fecal softeners (mineral oil), but the efficacy of these drugs is not long lasting and often has some adverse reactions. Acupoint massage therapy (AMT) is another traditional treatment technology in China applied to chronic constipation [4]. Because of its high efficacy and noninvasive characteristics, it has been widely used as a regimen to many diseases such as cancer, high blood pressure, and cerebral palsy.

The mechanism of AMT for functional constipation remains unclear. Recent studies have shown that gut microbiome plays a major role in the development and treatment of CFC [5–7]. In addition, some studies have shown that gut microbiome have certain efficacy in the adjuvant treatment of CFC [8]. There are various kinds of microorganisms living in the human gastrointestinal tract. The gut microbiome is combined according to a certain proportion. The bacteria restrict each other, depend on each other, and maintain a certain ecological balance. Gut microbiome participates in diverse physiological activities in the host, acting as a biological barrier to prevent the adhesion and invasion of pathogenic bacteria and potential pathogenic bacteria. Current studies have found that constipation is often accompanied by imbalance of gut microbiome, and imbalance of gut microbiome will have many adverse effects on the human body, such as producing multiple intestinal endotoxins, inducing colon cancer, accelerating aging, and promoting a variety of intestinal diseases [9–11]. Compared with the normal gut microbiome, the changes of gut microbiome in patients with CFC was mainly manifested in the relative decrease of specific anaerobic bacteria, such as *Lactobacillus*, *Bifidobacterium*, and *Bacteroides*, and the relative increase of potential pathogenic bacteria, such as *Pseudomonas aeruginosa*, *Campylobacter jejuni*, and *Clostridium putrefaciens* [12]. The changes of gut microbiome in CFC patients are different from each other, but most of the results show that the changes of gut microbiome are mainly due to the relative decrease of specific anaerobic bacteria such as *Lactobacillus* and *Bifidobacterium* and the relative increase of potential pathogenic bacteria such as *Clostridium putrefaciens*.

To investigate the clinical efficacy of AMT in the treatment of CFC on gut microbiome, we applied 16SrDNA sequencing analysis on the fecal of CFC patients with or without AMT. The significant difference was identified on the abundance of *Fusicatenibacter*, *Pseudobutyrivibrio*, and *Ruminiclostridium* which are novel findings in AMT treatment of CFC.

## 2. Materials and Methods

### 2.1. Patients

From May 2017 to May 2019, 104 patients with chronic functional constipation in the Second People's Hospital Affiliated to Fujian University of Traditional Chinese Medicine were selected as the research subjects, including 49 males and 55 females. During the intervention period, there were two abscission cases in the control group and one case in the experimental group. The average age was 49.04 ± 12.44 years and 52.74 ± 10.27 years, respectively, in experimental and control groups, and the duration of disease was 9.03 ± 4.49 and 9.64 ± 4.97 months (Table 1). Inclusion criteria were as follows: (1) conforming to chronic functional constipation IV diagnostic criteria (6)): (a) in the preceding 3 months, two or more of the following two cases occurred and at least 25% of them had difficulty in defecation. At least 25% of the defecation was dry manure or hard dung. At least 25% of the defecation cases were impatient. At least 25% of the defecation cases had anorectal obstruction and/or blockage. At least 25% of the defecation needed manual assistance. Fingers were used to assist defecation and pelvic floor support, fewer than three times a week. (b) Fewer cases of defecation occur without laxative. (c) Diagnosis of irritable bowel syndrome (IBS) is not reached; (2) clinical symptoms at least 6 months before diagnosis; (3) no antibiotics, probiotics, and drugs causing constipation in the past 3 months; (4) colonoscopy in the past 6 months. Colonoscopy before admission excluded intestinal organic lesions; (5) no other drugs were taken during the treatment of synbiotic; (6) synbiotics and other probiotics were not discontinued during the treatment. Exclusion criteria: (1) cardiovascular diseases, diabetes mellitus, tumors, and nervous system diseases; (2) expected outing plans during the trial; (3) body mass index (BMI) <16.0 kg/m^2^ or >30.0 kg/m^2^; (4) pregnancy or lactation; (5) usage of other probiotic foods or drugs one month before consultation; (6) taking anticholinergic drugs and antiabdominal drugs one month before consultation and laxatives and antibiotics; (7) history of gastrointestinal surgery, colorectal adenoma, and other diseases. All the subjects signed the informed consent, and this study was examined by the ethics committee of our hospital. The following indicators were assessed one week before intervention and two weeks after intervention.

### 2.2. Ethical Committee/Review Board

This study was approved by the Ethics Committee of the second people's Hospital Affiliated to Fujian University of traditional Chinese medicine. The date of the approval is 2019.4.19. The approval number is 2017-KL-003-01.

### 2.3. Main Symptoms of Clinical Constipation

Constipation symptoms including fecal sensation, frequency of self-defecation, degree of defecation exertion, time of defecation, incomplete defecation, and nature of feces (Bristol classification of feces: IV-VII, 0; III, 1; II, 2; I, 3) were assessed at admission, end of treatment, and 2 and 4 weeks after treatment. According to the symptoms, the scores were 0–3, respectively, and the total score was the sum of the above six items.

### 2.4. Quality of Life Assessment

The quality of life was assessed by the Mapi Research Trust authorized PAC-QOL Chinese Version Health Questionnaire (PAC-QOL). At the time of admission, at the end of treatment, and at the end of 2 and 4 weeks after treatment, the patients were assessed by filling in the form of psychological status, physiological status, satisfaction, and social relations.

### 2.5. Criteria for Judging Clinical Efficacy

For cured, constipation disappeared, and defecation interval time and the nature of feces were normal, the total score of symptoms decreased by more than 95%; markedly effective, the total score of symptoms decreased by more than 70%; effective, the total score of symptoms decreased by less than 70%, but more than 30%; ineffective, symptoms did not improve significantly, or even worsened, the total score of symptoms decreased by less than 30%.

### 2.6. Cytokines in Serum

For tumor necrosis factor alpha (TNF-*α*), interleukin-6 (IL-6), interleukin-8 (IL-8), interleukin-10 (IL-10), epidermal growth factor (EGF), vascular endothelial growth factor (VEGF), hypoxia inducible factor-1*α* (HIF-1*α*), and spleen tyrosine kinase (Syk), ELISA (MLBIO corporation, ml077385, ml059839, ml059840, and ml064299) was used for detection.

### 2.7. Extraction of DNA from Feces

1 g fecal sample was suspended with 10 mL sterile 0.1 mol/L sodium phosphate buffer for 15 min, centrifugation was performed at 200 × g for 10 min for 3 times, coarse particles were discarded, and the supernatant was collected; next, centrifugation at 9000 × g for 10 min, precipitate was collected and washed with 30 mL sodium phosphate buffer for 4 times, and finally 10 mL of phosphorus was suspended. Sodium buffer was then used to extract DNA from feces using QIAampDNA Stool MiniKit (QIAGEN, cat. no. 51504).

### 2.8. V3-V4 Sequencing of 16SrDNA

16SrDNA is located on the small ribosomal subunits of prokaryotic cells, including 10 conserved regions and 9 hypervariable regions. The conserved regions have little difference among bacteria, and the hypervariable regions have generic or species specificity, depending on their genetic relationship. There are some differences. Therefore, 16SrDNA can be used as a characteristic nucleic acid sequence to reveal biological species and is considered to be the most suitable indicator for bacterial phylogenetic and taxonomic identification. In this study, 16SrDNA amplicon sequencing was used to select the V3 V4 mutation region, and the conservative region was used to design universal primers as follows:

Forward_341 FTCGTCGGCAGCGTCAGATGTGTATAAGACAGCCTACGGNGGCWGCAG; reverse_785 RGTCTCGTGGCTCGGAGATGTGTAAGACAGGACTACHVGGTATCTAATCC was amplified by PCR and then the high-variable region was sequenced and identified. Based on Illumina HiSeq Sequencing Platform, a small fragment library was constructed using paired end method according to the characteristics of the amplified 16S region.

### 2.9. Sequencing Data Analysis

CleanTags were obtained by splicing and quality control of the original data obtained from Illumina HiSeq sequencing platform, and then chimeric filtering was performed to obtain effective data for subsequent analysis, namely, effective tags.

In order to study the diversity of species composition, effective tags of all samples were clustered, and the sequences were clustered into OTUs (operational taxonomic units) with 97% identity. Then, the representative sequences of OTUs were annotated. According to the OTU clustering results, on the one hand, species annotations are made on the representative sequences of each OTU, and the corresponding species information and species abundance distribution are obtained. The abundance, Alpha diversity calculation, and Venn diagram of OTUs were analyzed to obtain the common and specific OTU information of the exercise group and the less action group. The OTUs were aligned and phylogenetic trees were constructed, and the community structure differences between sport group and oligosport group were further obtained. Finally, PCoA analysis and demonstration are carried out. According to the species annotation results of OTUs, the top 10 species with the highest abundance in phylum, class, order, family and genus of each sample were selected, and the column accumulative map of relative abundance of species was generated to visualize the samples at different classification levels, species with higher relative abundance and their proportion. In order to further explore the difference of community structure between sport group and oligokinesis group, statistical analysis methods such as *T* test, MetaStat, LEfSe, Anosim, and MRPP were used to test the difference in species composition and community structure of grouped samples.

### 2.10. Statistical Analysis

Student's *t* double tail *t* test was used to compare the difference of clinical characteristics.

## 3. Results

### 3.1. Clinical Characteristics of Patients

The patients were randomly divided into a control group and an experimental group. During the intervention period, there were two abscission cases in the control group and one in the experimental group. The effective data were obtained in 101 cases, of which 51 cases were in the control group. There were 50 cases in the experimental group. There was no significant difference in gender, age, and duration between the two groups (*P* > 0.05) (Table 1). The total curative effect of the experimental group after 12 weeks was better than that of the control group (*P* < 0.05) (Table 2). There was no significant differences in PAC-QOL scores between the two groups before intervention (*P* > 0.05); after intervention, PAC-QOL scores were improved (*P* < 0.01), while those in the experimental group were lower than those in the control group (*P* < 0.05) (Table 2). After treatment, the main symptoms of constipation significantly improved (*P* < 0.05) in both groups, while the defecation inactivity, defecation time, and abdominal distension score were significantly lower in the experimental group than in the control group, as well as the defecation strain (Table 2).

### 3.2. Gut Microbiota Analysis of CFC Patients with or without AMT Treatment 

**Figure 1:**
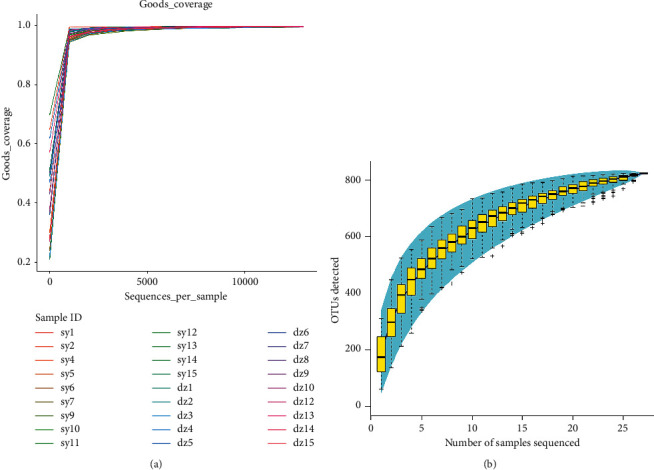
Rank abundance curves displaying the species richness and evenness in all samples. (a) Rarefaction curves with Good's coverage. (b) Specaccum.

To validate the effect of AMT treatment on microbiota diversity in CFC patients, the DNA from fecal samples of CFC patients with or without AMT treatment was carried out with pyrosequencing and statistical analysis. Clean tags in all the samples were distributed between 1969 and 41813. Clean tags were removed from chimeras to obtain valid tags (ultimately used for analysis) which ranged from 16705 to 36926 in all samples. The average length of valid tags is ranged from 423.27 to 433.15 bp. Subsequently, operational taxonomic unit (OUT) clustering and abundant statistics were performed for sequencing valid data. A total of 834 OTUs has averaged to 96.33% similarity score. The numbers of OUTs in each sample ranged from 62 to 310. All samples have a Good's coverage over 99.5%, suggesting that the depth of the gut microbiota is optimal for further analysis (Figure 1(a)). According to the rank abundance curves of each sample in experimental and control groups, the bacterial communities showed similar patterns (Figure 1(b)).

In order to compare the alpha diversity in each sample, the diversity index of each sample is counted. The Chao1 and Shannon diversity index suggested no significant difference in species richness and evenness between experimental and control group (Figure S1). Other estimators of alpha diversity such as PD whole tree, Simpson, and observed species also displayed no significant difference between two groups (Figure S1). The spectrum species accumulation curve is used to describe the increase of species with the increase of sampling. The spectrum species accumulation curve is widely used to estimate the adequacy of sampling amount and species richness in the biodiversity and community surveys. The results show that, when the sample size reaches 22, the curve tends to be flat, indicating that the sampling is sufficient and the subsequent species difference analysis can be carried out (Figure 1(b)).

**Figure 2:**
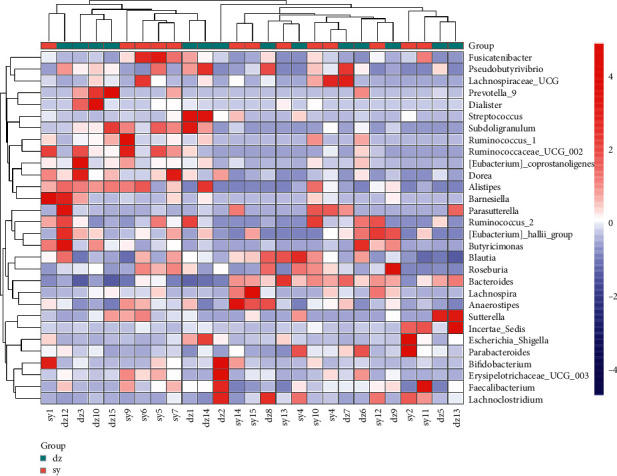
Heatmap showing the abundance of different top 30 microbial in all samples. The samples were clustered according to these relative abundances. The phylogenetic tress could not classify the samples from the experimental or control groups into the same clade.

In order to find out the species with more or less aggregation in each sample, we selected the top 30 genus according to the species annotation and abundance information of all samples at the genus level and clustered them from the two levels of species and samples according to their abundance information in each sample. As shown in the result, none of the bacterial genus aggregated more or less in all the cases of the experimental group or the control group, which indicates that the species abundance varies greatly between each case (Figure 2). If it is necessary in order to identify the structural differences between the experimental group and the control group, an overall analysis of each group is required.

To analyze the difference of OUT composition between experimental group and control group, we carried out a principal coordinate analysis (PCoA) based on weighted Unifrac distance. Through a series of eigenvalues and eigenvector sorting, the most important elements and structures are extracted from the multidimensional data, and the principal coordinate combination with the largest contribution rate (PC1 = 17.34%, PC2 = 7.32%, and PC3 = 6.08%) is selected for the graphic display. As shown in Figure S2, the samples between the two groups were divided into two clusters, indicating that the OTUs composition of intestinal flora between the two groups was different.

The above results indicate that similar levels of bacterial richness and diversity were found in the gut microbiota of CFC patients with or without AMT treatment. The overall structures of the gut microbiota also have not been significantly different.

### 3.3. Abundant Levels of Certain Bacteria Are Associated with AMT

**Figure 3:**
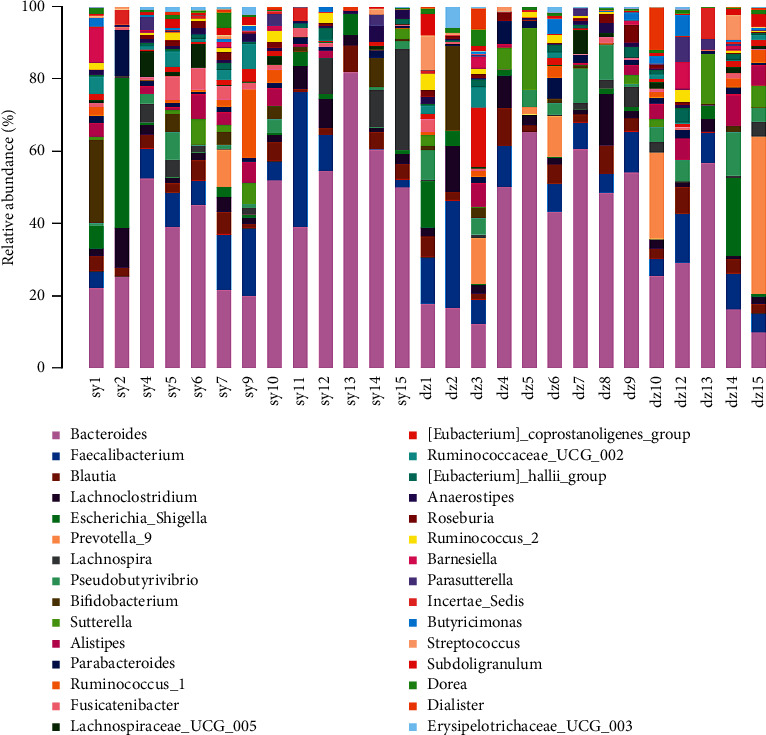
The 16S rRNA gene amplicon sequencing showing differences in the genus level between the samples from the experimental and control groups.

**Figure 4:**
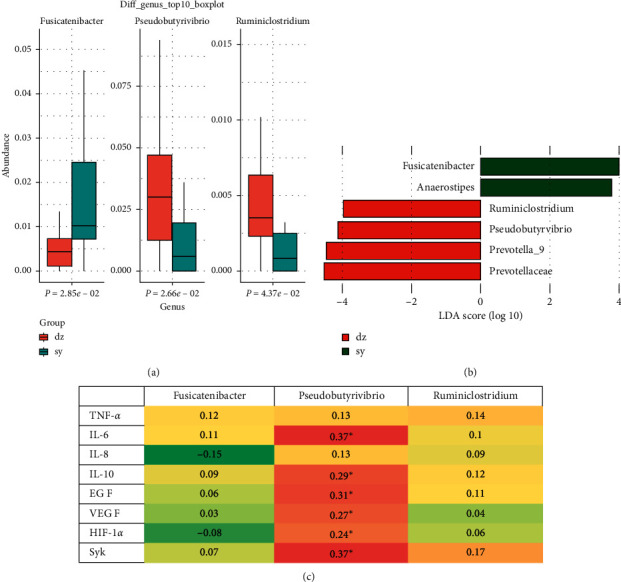
The difference analysis of specific species and genus. (a) Comparison of relative abundance of Fusicatenibacter, Pseudobutyrivibrio, and Ruminiclostridium between experimental and control groups. Data were presented as box plots with the average and range. The statistical significance was tested by MannWhitney, *P* < 0.05. Bar color indicates the group: red, experimental; blue, control. (b) LEfSe analysis of discriminative features in gut microbiome from CPC patients with or without AMT. The LDA level was set as LDA >3, *P* < 0.05. Bar color indicates the group: red, experimental; blue, control. (c) Spearman correlation analysis of cytokines, CFC-related protein, and the relative abundance of Fusicatenibacter, Pseudobutyrivibrio, and Ruminiclostridium.

The species composition of the top 30 taxonomic levels (phylum, class, order, family, genera, and species) with the highest relative abundance of species in the experimental group and the control group was analyzed (Figure 3). Based on the community abundance data obtained from the different analysis, the significance test of the difference between groups was carried out. Microbial families with different richness in the microbial community of the experiment and control groups could be detected by strict statistical methods, and the hypothesis test was carried out to evaluate the significance of the observed difference. According to ANOVA statistics, there were significant differences in genus taxonomy between the two groups (Figure 4(a)). The abundance of *Fusicatenibacter* in the experimental group was much significantly lower than that in the control group (*P*=0.0285) (Figure 4(a)). The abundance of *Pseudobutyrivibrio* and *Ruminiclostridium* in the experimental group was significantly higher than that in the control group (*P*=0.0266, *P*=0.0437) (Figure 4(a)).

To identify the specific bacterial taxa associated with AMT treatment, we compared the compositions of the fecal microbiota using the linear discriminant analysis effect size (LEfSe) method. A cladogram represents the structures of the fecal microbiota and the predominant bacteria in two groups, and the largest differences in the taxa between the two communities are comparable. In total, the LEfSe analysis revealed 6 discriminative features (linear discriminant analysis (LDA) >3, *P* < 0.05, Figure 4(b)) in the genus levels. Members of the *Ruminiclostridium, Pseudobutyrivibrio, Prevotell_9*, and *Prevotellaceae* were enriched in the experiment samples, whereas members of the *Fusicatenibacter* and *Anaerostipes* were enriched in the control samples. Therefore, these taxa may be used as biomarkers to discriminate two group patients.

### 3.4. Cytokines and CFC-Related Protein Are Correlated with Gut Microbial

Before and after intervention, the protein levels were determined by ELISA including TNF-a, IL-6, UL-8, IL-10, EGF, VEGF, HIF-1a, and Syk. There is no significant difference between control and experimental groups before intervention. All proteins were significantly reduced in experimental groups after intervention compared with that in the control group.

The relationships between the gut microbiota and cytokine-related proteins were explored. A correlation matrix based on Spearman correlation distance confirmed correlations among 8 protein levels in PBMC, and 3 genera showed significant differences between the two groups according to the ANOVA method (Figure 4(c)). The results revealed significant correlations between the abundance of Pseudobutyrivibrios and cytokines, including IL-6, IL-10, EGF, VEGF, HIF-1*α*, and Syk (Figure 4(c)).

### 3.5. KEGG Function Prediction

**Figure 5:**
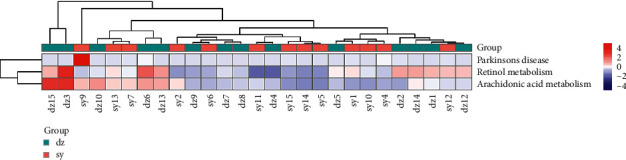
PICRUS analysis predicting the KEGG function of gut microbiome based on 16S sequence. The heat map displays the Kruskal–Wallis statistical results. The samples and microbiome were clustered in horizontal and vertical phylogenetic trees.

Furthermore, based on the 16S sequencing data annotated by Greengenes database, the function of PICRUS was used to predict and analyze the difference in microbiome function between different samples. Based on 16S KEGG function prediction, we can analyze gene protein sequences (KEGG Genes), endogenous and exogenous chemicals (KEGG Ligand), molecular interaction and metabolic pathway maps (KEGG Pathway), and hierarchical relationships among various organisms (KEGG Brite). The results showed that Parkinson disease, retinol metabolism, and arachidonic acid metabolism in the KEGG pathway could reflect the functional differences of intestinal flora between experimental group and control group (Figure 5).

## 4. Discussion

In this study, the Illumina HiSeq sequencing platform was used to analyze the structure of the gut microbiome community. The results showed that the composition of gut microbiome was quite different between the experimental group and the control group, indicating that there was an interaction between AMT treatment and gut microbiome in CFC. There is numerous evidence that revealed that physical therapy could modify gut microbiome in disease. In intestinal inflammation, exercise training could have an impact on gut immune cell and microbiota to regulate the interactions during an inflammatory insult [13]. Exercise training is also involved in the interaction between gut microbiota and metabolism in obesity [14]. However, studies on CKD patients showed exercise could reduce the oxidative stress and attenuated inflammation, without changing of gut microbiome [15]. Here, CFC patients displayed different gut microbiome after AMT, suggesting novel evidence that physical therapy could regulate gut microbiome in diseases.

In this study, the abundance of *Fusicatenibacter* in the experimental group was significantly reduced compared to the control group, whereas the abundance of *Pseudobutyrivibrio* and *Ruminiclostridium* in the experimental group was significantly induced. *Pseudobutyrivibrio* is a butyric acid-producing genus, which can produce abundant short-chain fatty acids (SCFAs). SCFAs could be against inflammatory through G protein-coupled receptors, to maintain the balance of the intestinal mucosal immune system that is a benefit for CFC. The study on constipation patients revealed that *Lactobacillus casei* strain Shirota intervention could increase the stool SCFA levels with an induction of *Pseudobutyrivibrio* abundance [16]. In obese mice, the abundance of *Pseudobutyrivibrio* and the content of cecum propionic acid are associated [17]. In growing pigs, the formation of SCFA was correlated with butyrate-producing bacteria such as *Pseudobutyrivibrio* [18]. Thus, the experimental group in the current study has increased *Pseudobutyrivibrio* which may generate more butyrate to attenuate intestinal inflammation.

Furthermore, Parkinson disease, retinol metabolism, and arachidonic acid metabolism pathways were predicted as KEGG function for gut microbiome in CPC with AMT treatment. So far, it is unclear how these pathways interacted with CPC. Recently, the study identified the arachidonic acid metabolism as biomarkers for constipation rats [19]. Researchers also found arachidonic acid metabolites were induced during chronic intestinal inflammation [20]. Thus, arachidonic acid metabolism has interaction with CPC. The underlying mechanism remains to be investigated.

## 5. Conclusion

AMT could change the composition of gut microbiome which is associated with cytokines in CFC patients.

## Figures and Tables

**Table 1:** The characteristics of patients.

	*n*	Gender		Age	Duration (month)
		Male	Female		
Control	51	20	31	58.77 ± 13.77	8.75 ± 8.36
Experimental	50	26	24	59.91 ± 15.44	10.05 ± 9.38

**Table 2:** The curative effect after 12 weeks.

	Control		Experimental		P1	P2
	Before	After	Before	After		
*n*	51		50		—	—
Cured	—	1	—	2	—	—
Markedly effective	—	4	—	7	—	—
Effective	—	28	—	32	—	—
Ineffective	—	18	—	9	—	—
Effective ratio	—	64.70%	—	82.00%	—	—
PAC-QOL	95.64 ± 15.51	81.64 ± 14.68	98.29 ± 17.23	70.69 ± 16.73	0.42	≤0.00
*T*	0.81		3.49		—	—
Defecation frequency	2.07 ± 1.26	1.23 ± 1.10	1.82 ± 1.33	0.81 ± 0.68	0.38	0.04
Defecation inactivity	2.47 ± 1.34	1.27 ± 0.82	2.82 ± 1.09	0.87 ± 0.73	0.19	0.02
Defecation strain	2.51 ± 1.15	1.42 ± 1.15	2.11 ± 1.33	0.98 ± 0.86	0.14	0.05
Abdominal distension	1.37 ± .29	0.79 ± 0.48	1.52 ± 1.22	0.59 ± 0.43	0.58	0.04
Defecation time	2.02 ± 1.17	1.43 ± 1.06	2.45 ± 1.20	1 ± 0.98	0.72	0.05

P1 : comparison between before or after treatment of the control group; P2 : comparison between the before or after treatment of the experimental group; ^*∗*^*P* < 0.05, ^*∗∗*^*P* < 0.01.

## Data Availability

All the data used to support this study are available within the article.

## Abbreviations

CFC: Chronic functional constipation
AMT: Acupoint massage therapy
IBS: Irritable bowel syndrome
BMI: Body mass index
OTUs: Operational taxonomic units
PCoA: Principal coordinates analysis
LEfSe: Linear discriminant analysis effect size
LDA: Linear discriminant analysis
SCFAs: Short-chain fatty acids.

## Supplementary Materials

Supplementary Materials Figure 1S: the rank abundance analysis. On the abscissa, the OTU is sorted from most to least according to the number of sequences it contains. For example, “500” represents the OTU with the 500th abundance in the sample. In the vertical coordinate, the relative abundance of the OTU, for example, “0.01” represents 0.01% and “0.1” represents 0.1%. Figure 2S: the PCA (3D) analysis. The horizontal and vertical axes represent two eigenvalues that can reflect the variance to the greatest extent. Each point in the figure represents a sample, and the same color is the same grouping and similar samples together.
