# Supplementary figures and images for: Acupoint Massage Therapy Alters the Composition of Gut Microbiome in Functional Constipation Patients
Source: Evid Based Complement Alternat Med. 2021 Jan 12;2021:1416236. doi: 10.1155/2021/1416236 (PMC7815419; doi:10.1155/2021/1416236)

Figure S1 . **The Rank Abundance analysis**


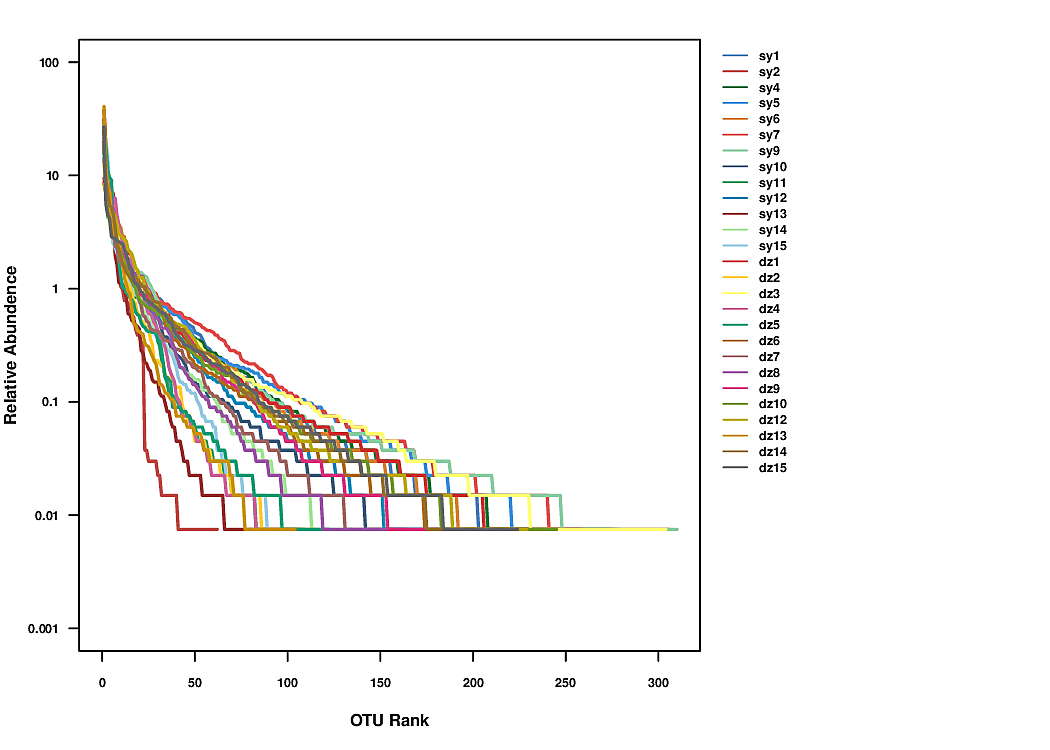


Figure S2 . **The PCA (3D) analysis**


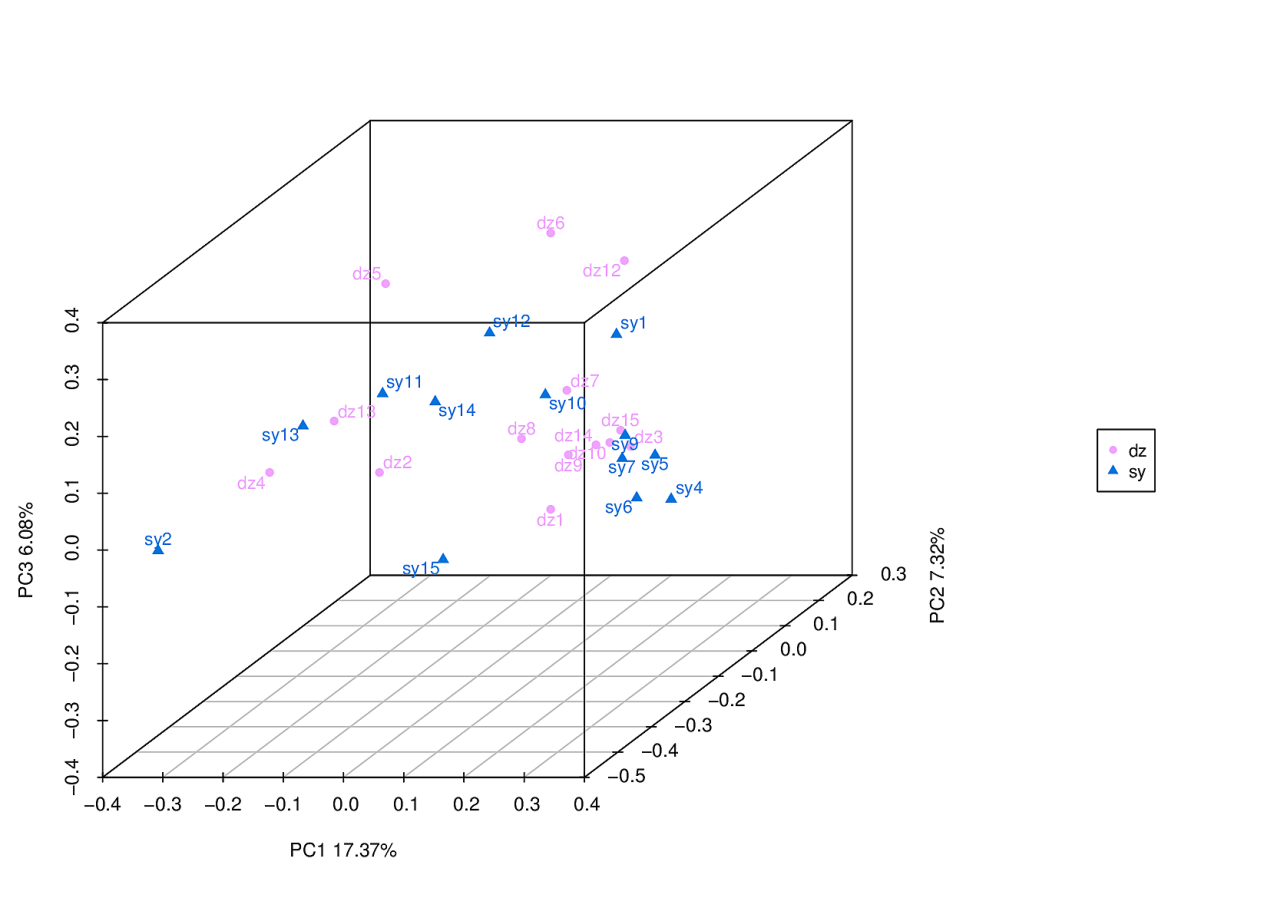

Supplement: Supplementary Materials — Figure 1S: the rank abundance analysis. On the abscissa, the OTU is sorted from most to least according to the number of sequences it contains. For example, “500” represents the OTU with the 500th abundance in the sample. In the vertical coordinate, the relative abundance of the OTU, for example, “0.01” represents 0.01% and “0.1” represents 0.1%. Figure 2S: the PCA (3D) analysis. The horizontal and vertical axes represent two eigenvalues that can reflect the variance to the greatest extent. Each point in the figure represents a sample, and the same color is the same grouping and similar samples together. [file 1416236.f1.docx]
